# Causal Interplay Between Inflammatory Cytokines and Lipid Metabolites in Serous Ovarian Carcinoma: Insights From a Genetic Association Study

**DOI:** 10.1002/jcla.70250

**Published:** 2026-05-08

**Authors:** Chong‐ze Yang, Lan‐hui Qin, Cheng‐can Huang, Xiao‐li Huang, Ying‐xia Yang, Wei Lan, Miao Fan

**Affiliations:** ^1^ Department of Radiology, Guangxi Hospital Division of the First Affiliated Hospital Sun‐Yat‐Sen University Nanning Guangxi Zhuang Autonomous Region People's Republic of China; ^2^ Department of Radiology The First Affiliated Hospital of Guangxi Medical University Nanning Guangxi Zhuang Autonomous Region People's Republic of China; ^3^ Department of Neurology Affiliated Hospital of Youjiang Medical University for Nationalities Baise Guangxi Zhuang Autonomous Region People's Republic of China; ^4^ School of Computer, Electronic and Information Guangxi University Nanning Guangxi Zhuang Autonomous Region People's Republic of China; ^5^ Department of Radiology First Affiliated Hospital of Sun Yat‐Sen University Guangzhou Guangdong Province People's Republic of China

**Keywords:** blood metabolites, inflammatory cytokines, mediation analysis, Mendelian randomization, serous ovarian carcinoma

## Abstract

**Background:**

The causal roles and interactions of inflammatory cytokines and metabolic reprogramming in serous ovarian carcinoma (SOC) remain unclear. This study explored their relationships using Mendelian randomization (MR).

**Methods:**

In this two‐sample MR analysis, GWAS data of inflammatory cytokines and blood metabolites were used as exposures, and SOC from the FinnGen consortium served as the outcome. Inverse variance weighted (IVW) was the primary MR method, supplemented by MR Egger, weighted median, simple mode, and weighted mode. Two‐step mediation MR was applied to evaluate whether specific metabolites mediated the effect of inflammatory cytokines on SOC. Sensitivity analyses, including heterogeneity and pleiotropy tests, were conducted to assess robustness.

**Results:**

Five inflammatory cytokines were identified as risk factors for SOC: CSF1 (OR = 1.69, 95% CI: 1.17–2.43), CXCL1 (OR = 1.40, 95% CI: 1.01–1.93), IL‐20 (OR = 1.86, 95% CI: 1.03–3.35), IL‐8 (OR = 1.61, 95% CI: 1.08–2.39), and VEGF‐A (OR = 1.24, 95% CI: 1.00–1.54). Furthermore, 1‐Palmitoyl‐GPG (16:0) potentially mediates the relationship between IL‐8 and SOC, explaining ~10% of the total effect. No pleiotropy or heterogeneity was detected.

**Conclusions:**

This two‐sample MR study provides preliminary genetic evidence that inflammatory cytokines contribute to SOC risk, with lipid metabolism partially mediating IL‐8 effects. These findings highlight the interplay between inflammation and metabolism in SOC pathogenesis and suggest potential biomarkers and therapeutic targets. Due to limited sample sizes and European‐only ancestry datasets, these findings require validation in larger, multi‐ancestry cohorts.

## Graphical abstract

This study utilizes a two‐sample Mendelian randomization framework to establish a causal link between circulating inflammatory proteins, blood metabolites, and the risk of serous ovarian carcinoma. Our findings identify Interleukin‐8 as a significant risk factor and demonstrate that 1‐Palmitoyl‐GPG (16:0) acts as a metabolic mediator within this pathway. These results provide genetic evidence for the interplay between inflammation and lipid metabolism, offering novel laboratory biomarkers for the early assessment of ovarian cancer susceptibility.

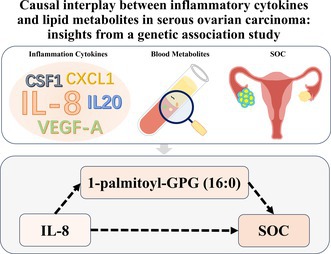

**Abbreviations**
GWAS: genome‐wide association study
IVs: instrumental variables
IVW: inverse variance weighted
MR: mendelian randomization
OR: odds ratios
SOC: serous ovarian carcinoma

## 1 Background

Ovarian cancer remains a leading cause of tumor‐related mortality among women worldwide. Among its histological subtypes, serous ovarian carcinoma (SOC)—particularly high‐grade serous ovarian carcinoma (HGSOC)—is the most aggressive form and is associated with the poorest prognosis [1, 2]. Due to the absence of specific symptoms in the early stages and the lack of effective population‐based screening strategies, the majority of patients are diagnosed at an advanced stage [3]. Moreover, the high recurrence rate and the development of acquired chemoresistance further contribute to the unfavorable overall survival outcomes. Previous studies have suggested that the occurrence of SOC is associated with a family history of endometriosis, environmental exposures, and adverse lifestyle factors [4]. However, the precise pathogenesis of SOC remains largely unclear. In the current era of precision and personalized medicine, identifying actionable causal targets and translatable biomarkers from etiological and pathway perspectives holds considerable scientific value and clinical significance.

Chronic inflammatory microenvironments are widely recognized as major drivers of disease initiation and progression, with inflammatory cytokines and chemokines playing pivotal regulatory roles within these processes [5]. Multiple mediators contribute to this pathological state by promoting angiogenesis, inducing epithelial–mesenchymal transition, reprogramming myeloid cells, and recruiting immunosuppressive cell populations, thereby sustaining a “pro‐tumorigenic inflammation–immune imbalance” that facilitates disease development [6, 7]. Importantly, inflammation‐related signaling pathways do not operate in isolation or arise from a single causal event; rather, they are tightly coupled and interact dynamically with metabolic networks [8, 9].

Parallel to inflammation, metabolic reprogramming is also recognized as a hallmark of cancer [10, 11]. Blood metabolites not only reflect the status of energy metabolism and biosynthetic pathways but may also serve as critical bridges linking chronic inflammation to malignant transformation [12]. Observational studies have provided foundational evidence linking specific metabolomic profiles to ovarian cancer. For instance, using a two‐step metabolomics strategy, Chen et al. identified specific serum metabolites, most notably 27‐nor‐5β‐cholestane‐3,7,12,24,25 pentol glucuronide (CPG), as potential diagnostic biomarkers for epithelial ovarian cancer, highlighting the presence of metabolic disruptions even at early disease stages [13]. Furthermore, a 23‐year prospective study by Zeleznik et al. demonstrated that elevated pre‐diagnostic circulating levels of specific lipid groups, particularly sphingomyelins (SMs), are prospectively associated with an increased risk of ovarian cancer [14]. However, these findings are inherently limited by reverse causation and confounding biases, making it difficult to establish causal inference. Consequently, distinguishing between concomitant alterations and true causal relationships, and quantifying their mediating contributions to disease mechanisms, remains a major challenge in aetiological research and therapeutic target discovery.

Because of these limitations in traditional observational designs, determining whether inflammatory and metabolic alterations are causes, consequences, or mere bystanders of SOC requires alternative methodological approaches. While randomized controlled trials (RCTs) are the gold standard for establishing causality, they often face ethical and feasibility constraints when addressing etiological questions. Mendelian randomization (MR), which employs genetic variants associated with exposures as instrumental variables (IVs), provides a robust strategy to mitigate confounding and reverse causation, thereby offering a potential solution to these limitations. Based on the principles of Mendel's law of segregation and independent assortment, genetic variants are randomly allocated during gamete formation and are fixed at conception. These genetic assignments precede disease onset and are generally independent of self‐selected environmental or lifestyle factors; MR is highly resilient to reverse causation and unmeasured confounding [15, 16]. Furthermore, two‐step mediation MR allows the dissection of the sequential pathway from exposure to mediator to outcome, enabling quantification of the mediator's causal contribution and elevating associations to causal evidence. With the increasing availability of large‐scale, publicly accessible resources such as FinnGen and the NHGRI‐EBI GWAS catalog, the feasibility and reproducibility of these strategies are now greatly enhanced.

Building on these considerations, the present study was designed to test two hypotheses: (1) inflammatory cytokines exert a causal effect on the risk of SOC; and (2) specific blood metabolites mediate the relationship between inflammatory cytokines and SOC. To this end, we applied a two‐sample MR framework to investigate the potential causal associations between inflammatory cytokines and SOC, followed by a two‐step mediation MR approach to evaluate whether particular metabolites serve as intermediates in this pathway. Our goal was to further elucidate the mechanistic axis linking inflammation, metabolism, and SOC, thereby providing novel, testable clues for mechanistic understanding, risk stratification, and biomarker development, while laying the foundation for subsequent functional validation and translational research.

## 2 Methods

### 2.1 Study Design Overview

**FIGURE 1:**
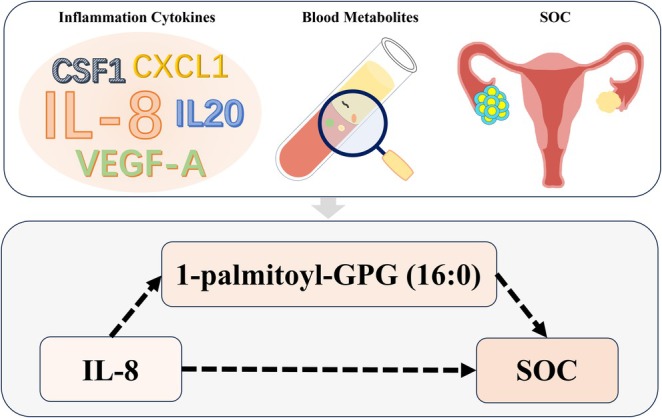
The flowchart of this study.

In this study, we employed a two‐step MR approach to investigate the potential causal associations between inflammatory cytokines and SOC, and to further assess whether these associations are mediated by blood metabolites. In the first step, a two‐sample MR analysis was conducted to evaluate the causal relationships of inflammatory cytokines and blood metabolites with SOC, thereby identifying candidate exposures associated with SOC risk. In the second step, we examined the potential causal relationships between the selected inflammatory cytokines and metabolites, and subsequently quantified the mediating effects of metabolites on the inflammatory cytokines–SOC pathway (Figure 1). This study was reported in accordance with the STROBE‐MR (Strengthening the Reporting of Observational Studies in Epidemiology using Mendelian Randomization) guidelines (Data S3).

The primary analytic strategy of this study was two‐sample MR. MR analysis relies on three core assumptions to ensure unbiased causal inference: (1) the IVs are strongly associated with the exposure of interest; (2) the IVs are independent of potential confounders; and (3) the IVs influence the outcome exclusively through the exposure, rather than through alternative pathways. All data used in this study were obtained from publicly available datasets; therefore, no additional ethical approval or informed consent was required.

### 2.2 Data Source

Genome‐wide association study (GWAS) summary data for ovarian cancer were obtained from the FinnGen consortium (https://www.finngen.fi/en/access_results). The dataset included 1025 cases of ovarian cancer, of which 852 were SOC, and 167,189 controls. For the present analysis, we specifically utilized the GWAS summary statistics derived from the case–control comparison of SOC (852 cases versus 167,189 controls) to perform MR analyses.

The GWAS summary statistics for 91 inflammatory cytokines (GCST90274758–GCST90274848) [17] and for 1400 blood metabolites and metabolite ratios (GCST90199621–GCST902010209) [18] were downloaded from the NHGRI‐EBI GWAS Catalog (https://www.ebi.ac.uk/gwas/).

### 2.3 Instrumental Variable Selection

Single nucleotide polymorphisms (SNPs) significantly associated with the exposures of interest were selected as IVs, using a threshold of *p* < 5 × 10^−5^. Several quality control procedures were applied to ensure the validity of IVs. First, linkage disequilibrium (LD) pruning was performed with a threshold of *r* ^2^ < 0.001 and a clumping window size of 10,000 kb. Second, the F‐statistic for each SNP was calculated using the formula F = β^2^/SE^2^. SNPs with an F‐statistic ≥ 10 were considered sufficiently strong instruments, whereas those with F < 10 were regarded as weak instruments and excluded from subsequent analyses.

### 2.4 Statistical Analysis

A two‐step MR analysis was conducted to estimate the potential causal relationships of the exposure (inflammatory cytokines) and the mediator (metabolites) with the outcome (SOC). This approach was applied to assess the extent to which metabolites mediated the exposure–outcome relationship. Specifically, the causal effect of the exposure on the mediator (β₁) was first estimated, followed by the effect of the mediator on the outcome (β₂). The mediation effect was then calculated as β₁ × β₂, and the proportion of the mediation effect relative to the total effect was quantified using the delta method (Figure 1).

MR analyses were performed using five methods: inverse‐variance weighted (IVW), MR‐Egger, weighted median, simple mode, and weighted mode. Among these, IVW was considered the primary analytic approach; therefore, *p*‐values derived from the IVW method were regarded as the main results, with the other four methods serving as sensitivity analyses. Results were reported as odds ratios (ORs) with corresponding 95% confidence intervals (CIs), and a *p* < 0.05 was considered statistically significant. To ensure the causal direction was from exposure to outcome, reverse MR analyses were additionally conducted. Only associations with significant *p*‐values in the forward MR analysis and nonsignificant *p*‐values in the reverse MR analysis were considered robust; results failing to meet these criteria were excluded from further interpretation.

To further ensure the robustness of causal inferences in the two‐sample MR framework, we conducted a series of sensitivity analyses. Heterogeneity across SNP‐specific causal estimates was assessed using Cochran's Q test, with a *p* < 0.05 indicating significant heterogeneity. Horizontal pleiotropy was evaluated using the MR‐Egger regression intercept and the MR‐PRESSO global test; associations were considered to exhibit horizontal pleiotropy if either test yielded a *p* < 0.05. Causal relationships with evidence of horizontal pleiotropy were deemed unreliable and excluded from further interpretation. After testing for pleiotropy and heterogeneity, we performed leave‐one‐out analyses for eligible exposures. In this approach, each SNP was sequentially excluded, and the combined effect of the remaining SNPs was recalculated. If the overall confidence interval showed no substantial changes after removing individual SNPs, the result was considered robust.

All statistical analyses were performed using R software (version 4.2.1), with the TwoSampleMR package applied for MR analyses.

## 3 Results

### 3.1 Overview

Through MR analysis, we identified five inflammatory cytokines and 68 blood metabolites with potential causal associations with SOC. Mediation MR analysis further revealed that 1‐palmitoyl‐GPG (16:0) mediated the effect of IL‐8 on SOC (Data S1 and S2).

### 3.2 Effect of Inflammatory Cytokines on SOC

IVW analyses identified five inflammatory cytokines with significant causal associations with SOC, with the number of SNPs used in the MR analyses ranging from 8 to 19.

**FIGURE 2:**
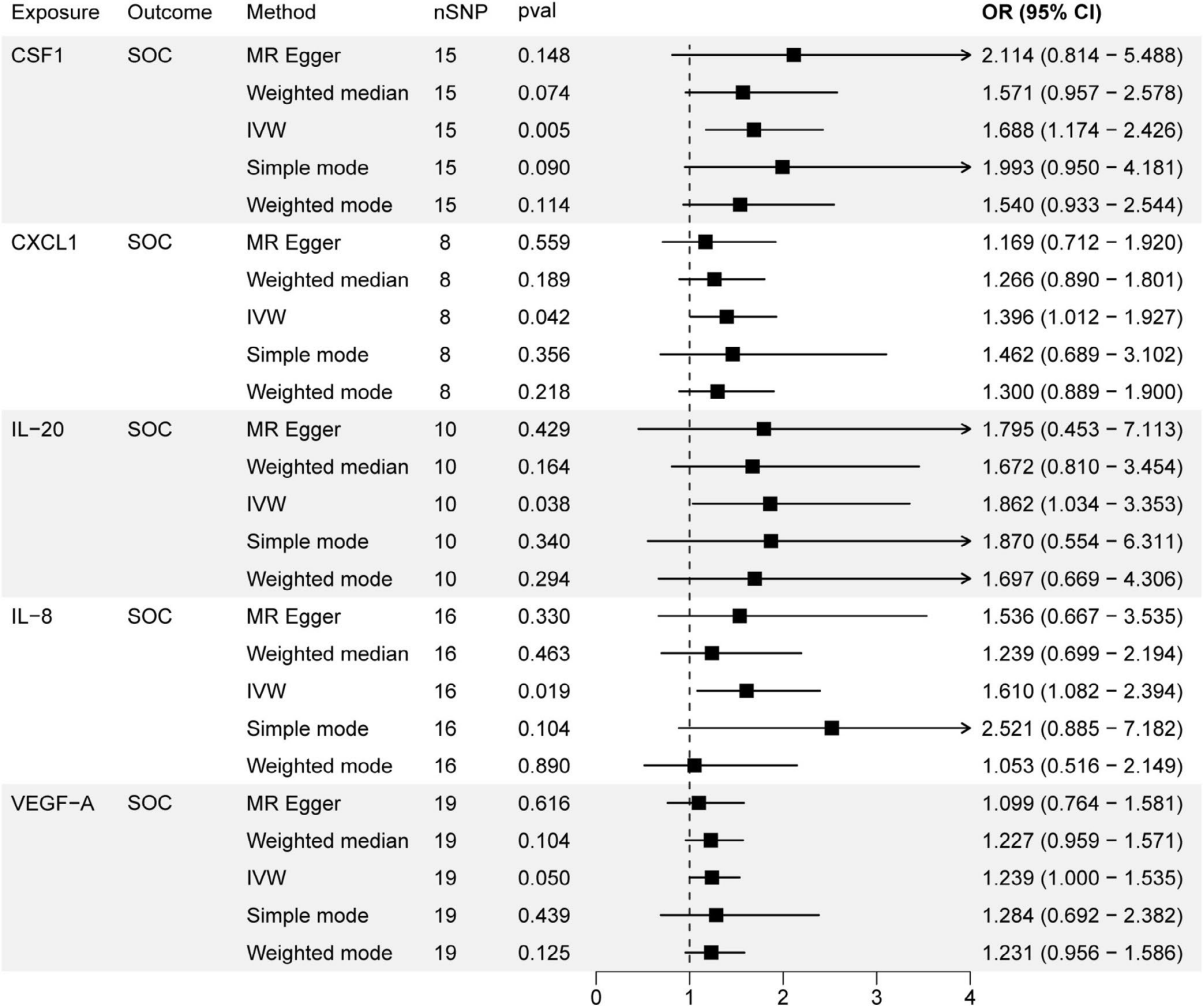
Forest plot of the causal relationship between circulating inflammatory proteins and SOC. IVW: Inverse variance weighted; nSNP: Number of single nucleotide polymorphisms; OR: Odds ratio.

**FIGURE 3:**
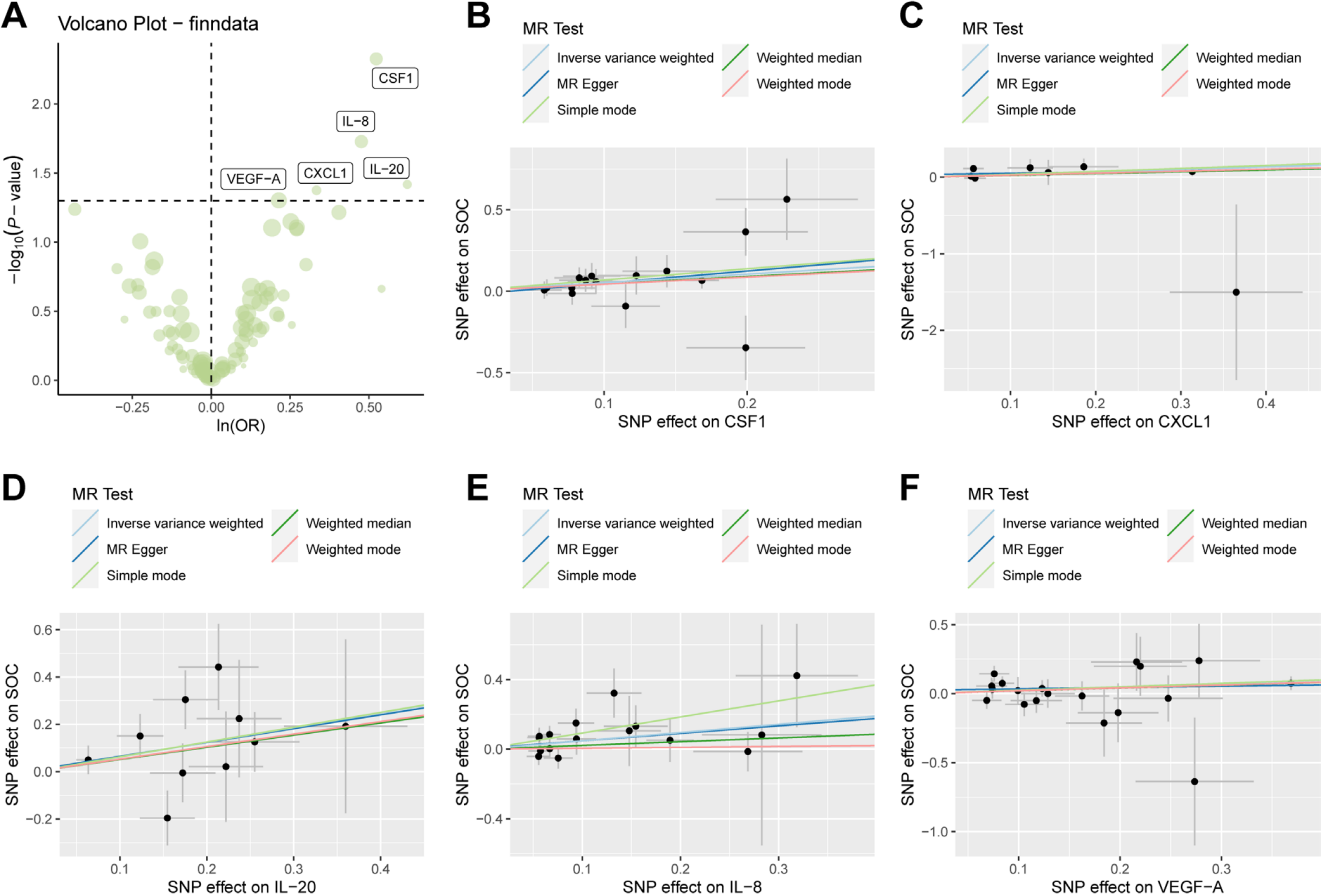
(A) Volcano diagram: Inflammatory cytokines with potential causal relationship with SOC. (B–F) Scatter plots for the causal association between CSF1, CXCL1, IL20, IL8, VEGF‐A, and SOC.

All five inflammatory cytokines were identified as risk factors for SOC. The IVW estimates were as follows: CSF1 (OR = 1.688, 95% CI: 1.174 to 2.426; F‐statistics: 20.88 to 203.67), CXCL1 (OR = 1.396, 95% CI: 1.012 to 1.927; F‐statistics: 20.89 to 547.00), IL‐20 (OR = 1.862, 95% CI: 1.034 to 3.353; F‐statistics: 20.97 to 27.03), IL‐8 (OR = 1.610, 95% CI: 1.082 to 2.394; F‐statistics: 21.10 to 61.76), and VEGF‐A (OR = 1.239, 95% CI: 1.000 to 1.535; F‐statistics: 21.08 to 943.51) (Figures 2 and 3). All F‐statistics significantly exceeded the conventional threshold of 10, indicating the absence of weak instrument bias. Furthermore, post hoc power calculations revealed varying levels of statistical power for these identified factors. CSF1 demonstrated adequate statistical power (80.8%, with a minimum detectable OR of 1.68 required to achieve 80% power). IL‐8 and IL‐20 achieved robust power approaching the standard threshold (73.9% and 73.1%, respectively). In contrast, due to their more modest effect sizes and the limited sample size of SOC cases, the analyses for CXCL1 and VEGF‐A were relatively underpowered (30.5% and 29.4%, respectively), suggesting that these specific associations should be interpreted with caution.

### 3.3 Effect of Blood Metabolites on SOC

MR analyses identified a total of 68 blood metabolites with significant causal associations with SOC, with the number of SNPs used in the analyses ranging from 14 to 46 (F‐statistics: 19.503 to 2297.785). Among these, 39 metabolites were positively associated with SOC risk, while 29 metabolites were negatively associated (Data S2).

### 3.4 Effect of Inflammatory Cytokines on Blood Metabolites

**FIGURE 4:**
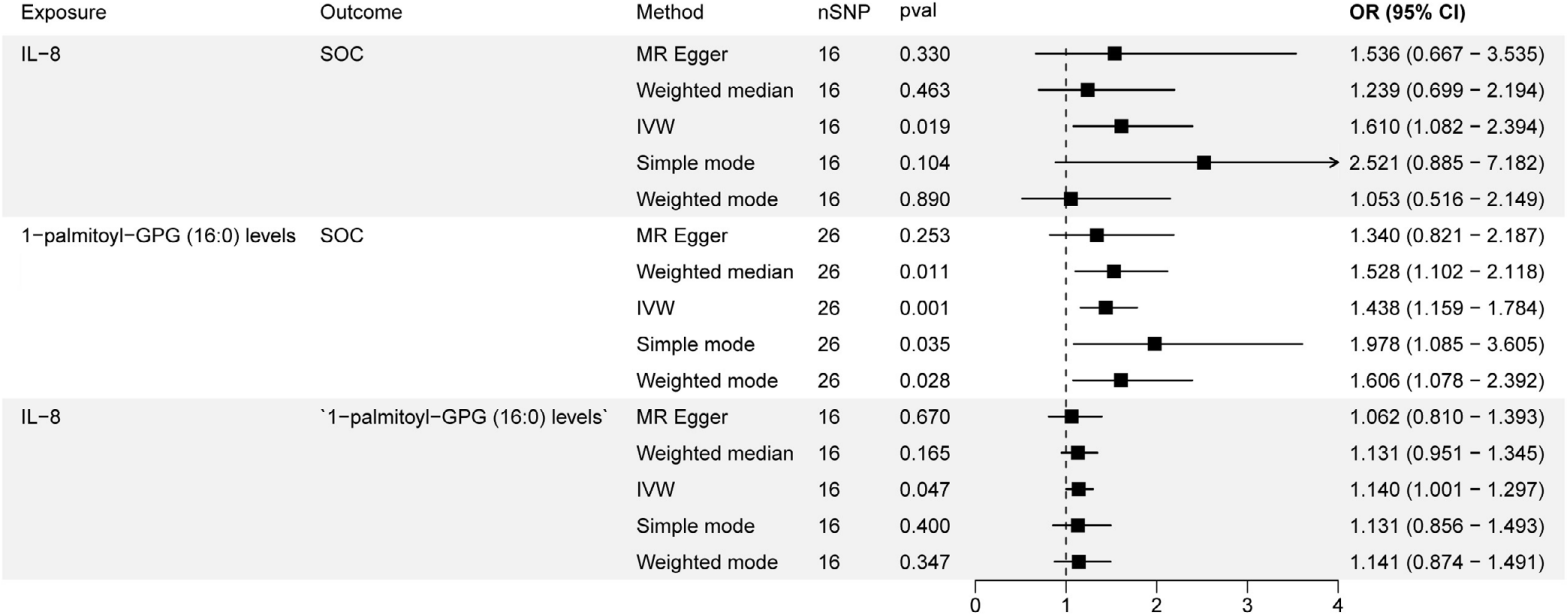
Forest plot of the causal relationship between IL‐8, 1‐Palmitoyl‐GPG (16:0), and SOC. IVW: Inverse variance weighted; nSNP: Number of single nucleotide polymorphisms; OR: Odds ratio.

In this study, MR analyses identified five inflammatory cytokines and 68 blood metabolites with potential causal associations with SOC. We subsequently investigated the causal relationships between these five inflammatory cytokines and the 68 metabolites. IVW analysis revealed suggestive evidence for a positive association between IL‐8 and 1‐palmitoyl‐GPG (16:0) (OR = 1.140, 95% CI: 1.001–1.297), based on 16 SNPs used in the MR analysis (Figure 4).

### 3.5 Mediation Effect of 1‐Palmitoyl‐GPG (16:0) in the Causal Association Between the IL8 and SOC

Mediation MR analysis revealed that 1‐palmitoyl‐GPG (16:0) potential partially mediated the effect of IL‐8 on SOC. Specifically, IL‐8 exhibited a positive causal association with SOC (β = 0.4760, 95% CI: 0.0791–0.8729), and 1‐palmitoyl‐GPG (16:0) was similarly associated with an increased risk of SOC (β = 0.3633, 95% CI: 0.1475–0.5791). Furthermore, a positive association was observed between IL‐8 and 1‐palmitoyl‐GPG (16:0) levels (β = 0.1307, 95% CI: 0.0015–0.2599). The indirect effect mediated by 1‐palmitoyl‐GPG (16:0) was estimated at 0.0475 (95% CI: −0.0073 to 0.1022), accounting for 9.98% of the total effect (Figures 1 and 4).

### 3.6 Sensitivity Analysis

Cochran's Q test indicated no significant heterogeneity among the selected SNPs. Neither the MR‐Egger regression nor the MR‐PRESSO global test detected evidence of horizontal pleiotropy. Furthermore, leave‐one‐out analysis demonstrated that the observed causal relationships were not driven by any single SNP.

## 4 Discussion

In this study, we applied bidirectional MR to screen 91 inflammatory cytokines and identified five with suggestive causal associations with SOC. Furthermore, we employed two‐step mediation MR to examine whether blood metabolites mediated the effects of inflammatory cytokines on SOC. The results demonstrated that CSF1, CXCL1, IL‐20, IL‐8, and VEGF‐A were suggestive risk factors for SOC. Mediation MR analysis further indicated that the lipid metabolite 1‐palmitoyl‐GPG (16:0) partially mediated the effect of IL‐8 on SOC. Multiple sensitivity tests confirmed the robustness of these findings. Collectively, this biomarker‐oriented approach provides novel and reliable insights into the etiology of SOC. To further elucidate the underlying mechanisms, we discuss the implications of these findings in the following sections.

Chronic inflammation and immune imbalance constitute a central background for the initiation and progression of SOC [19]. CXCL1, IL‐8, and IL‐20 belong to the chemokine family, which sustain and amplify inflammatory responses by binding to their respective receptors, CXCR1/2 or IL‐20R. Persistently elevated proinflammatory signaling leads to continuous activation of pathways such as NF‐κB and STAT3 within the ovarian cancer microenvironment, thereby shortening the G1 phase, accelerating DNA synthesis and cell division, promoting rapid tumor growth, and increasing genomic instability [20, 21]. CXCL1, through CXCR2, recruits immune cells and reprograms them toward immunosuppressive phenotypes, resulting in Arg‐1/iNOS/ROS‐mediated inhibition of CD8^+^ T cells and the expansion of Tregs [22, 23]. Elevated IL‐8 is also closely associated with neutrophil infiltration; high neutrophil levels, partly activated through Jagged2 signaling, impair CD8^+^ T‐cell cytotoxicity, further exacerbating chronic inflammation and immune evasion [24]. Clinically, serum CXCL1 levels are higher in patients with advanced ovarian cancer compared with those in early‐stage disease [25]; patients with elevated IL‐8 are more prone to metastasis and experience poorer overall survival; and animal studies have shown that blockade of IL‐8 signaling can reverse resistance to cisplatin and paclitaxel [26]. As a proinflammatory member of the IL‐10 family, IL‐20 not only contributes to the chronic inflammatory milieu but also promotes epithelial–mesenchymal transition upon receptor complex binding, thereby enhancing cell migration and invasion [27, 28]. Collectively, these findings suggest that inflammatory chemokines drive the disease course and malignant progression of SOC through chronic inflammation and its associated mechanisms.

The polarization of tumor‐associated macrophages (TAMs) represents another key mechanism in the progression of SOC. CSF1, a classical macrophage colony‐stimulating factor, binds to CSF1R and activates downstream PI3K–AKT, MAPK/ERK, and JAK/STAT3 signaling pathways, thereby promoting monocyte recruitment and differentiation into immunosuppressive M2‐type TAMs [29]. M2‐polarized TAMs secrete effector molecules such as VEGF and IL‐10, which act synergistically to promote angiogenesis and stromal remodeling. In addition, they impair antigen presentation and upregulate PD‐L1 expression, further weakening antitumor immune responses and fostering an immunosuppressive microenvironment that facilitates tumor progression and metastasis [30, 31]. Notably, CSF1 signaling complements the CXCL1–IL‐8 axis in immune cell recruitment by mobilizing myeloid‐derived suppressor cells (MDSCs). Together with TAMs, this forms a cooperative network that enhances tumor cell invasion, adhesion, and immune evasion [32].

Angiogenesis and hypoxia‐driven microenvironmental remodeling are hallmarks of tumor progression. VEGF‐A, a classical angiogenic factor, promotes endothelial cell proliferation, migration, and vascular permeability, thereby driving neovascularization in tumors [33]. VEGF‐A does not act in isolation; recent studies have shown that IL‐8 and IL‐20 can stimulate VEGF expression, exerting strong synergistic effects with VEGF‐A in angiogenesis. On the one hand, VEGF‐A activates the PI3K/Akt/mTOR and MAPK/ERK pathways to enhance endothelial cell migration and proliferation, accelerating vascular network formation. On the other hand, it promotes cytoskeletal reorganization and vascular permeability through the PLCγ/PKC–Src/FAK axis. Together, these mechanisms reinforce neovascular network construction, providing favorable conditions for tumor initiation and progression [34, 35]. The mismatch between rapid neovascularization and high metabolic demand creates a hypoxic tumor microenvironment, under which HIF‐1α becomes stabilized and binds to the VEGF‐A promoter, establishing a vicious cycle of hypoxia‐induced angiogenesis [36, 37]. Moreover, ovarian cancer cells themselves can express VEGFR‐2, forming an autocrine VEGF‐A/VEGFR‐2 signaling loop that further enhances invasive capacity [38, 39]. Clinically, high VEGF expression in serum or tumor tissue is closely associated with aggressiveness and metastatic potential. Patients with elevated VEGF levels typically exhibit shorter progression‐free intervals and overall survival, particularly in those with residual disease after surgery [40]. These findings indicate that VEGF not only functions as a key driver of angiogenesis and tumor progression but also serves as an important biomarker for prognostic stratification.

Blood metabolites may mediate the effects of inflammatory cytokines on SOC. Our two‐step mediation MR analysis suggested that the lipid metabolite 1‐palmitoyl‐GPG (16:0) potentially mediated the effect of IL‐8 on SOC. Given that this mediation effect reached nominal significance but was not robust to multiple testing correction, it should be interpreted as a hypothesis‐generating finding. This finding indicates that the pro‐tumorigenic effect of IL‐8 may not solely depend on chronic inflammation and immune evasion but may also be linked to lipid metabolic pathways. However, current evidence specifically addressing this metabolite remains limited. Abnormal abundance of 1‐palmitoyl‐GPG (16:0) reflects lipid metabolic reprogramming in ovarian cancer cells, with phospholipase A2 (PLA2) activity serving as a major pathway connecting 1‐palmitoyl‐GPG (16:0) to IL‐8 signaling [41, 42]. PLA2 generates various lysophospholipids, including 1‐palmitoyl‐GPG (16:0), which potentially amplify and sustain IL‐8 signaling in SOC, forming a metabolism–inflammation cascade [43, 44]. Similarly, a recent MR study reported that 1‐palmitoyl‐GPG (16:0) acts as a regulatory mediator of immune cell function in the pathogenesis of diabetes [45].

Collectively, our findings provide evidence that inflammatory cytokines may exert causal roles in the initiation and progression of SOC, with blood metabolites mediating part of these effects. This discovery offers novel molecular insights into the etiology of SOC, supporting the identification and development of more targeted biomarkers, and laying the foundation for new therapeutic strategies directed at inflammation, immunity, metabolism, and angiogenesis, thereby advancing personalized and precision medicine for SOC.

Nevertheless, several limitations should be acknowledged. First, the datasets employed in this study were primarily derived from European populations; thus, the generalizability of our results to other ancestries requires further validation. Second, the number of SNPs available for MR analyses remained relatively limited, and the datasets did not comprehensively cover all inflammatory cytokines or metabolites, which may have led to underestimation or omission of relevant mediation effects. Third, to ensure an adequate number of SNPs for these exposures, a relatively lenient significance threshold was utilized for IVs selection. While necessary, this relaxed threshold inherently carries a potential risk of introducing horizontal pleiotropy or weak instrument bias. Post hoc power calculations for the identified cytokines revealed a statistical power range of 29.4% to 80.8%, indicating that the analysis for factors with modest effect sizes may be underpowered and susceptible to false‐negative findings. Future studies should aim to replicate these findings in larger, multi‐ancestry cohorts and leverage integrative multi‐omics approaches, including single‐cell sequencing, to dissect the dynamic remodeling of the SOC tumor microenvironment. Such efforts will be critical for exploring the translational potential of these mechanisms in early diagnosis, risk stratification, and therapeutic decision‐making.

## 5 Conclusions

This study, based on a bidirectional two‐sample MR framework, systematically evaluated the potential causal relationships between inflammatory cytokines and SOC, and further explored the regulatory role of blood metabolites through two‐step mediation analysis. The results demonstrated that CSF1, CXCL1, IL‐20, IL‐8, and VEGF‐A are potential risk factors for SOC, while the lipid metabolite 1‐palmitoyl‐GPG (16:0) mediates the association between IL‐8 and SOC. These findings highlight the critical interplay among inflammation, immunity, metabolism, and angiogenesis in SOC pathogenesis, providing novel genetic evidence for disease etiology and laying a foundation for future precision interventions targeting inflammatory and metabolic pathways. Nevertheless, considering the limited statistical power due to the small case sample size and the restriction to European ancestry, future studies with larger, multi‐ancestry cohorts are essential to validate these findings.

## Author Contributions

Chong‐ze Yang and Lan‐hui Qin designed the study and drafted the manuscript. Chong‐ze Yang, Lan‐hui Qin, and Wei Lan performed data analysis. Cheng‐can Huang, Xiao‐li Huang, and Ying‐xia Yang prepared the figures. Miao Fan critically revised the manuscript. All authors read and approved the final version of the manuscript.

## Funding

This work was supported by National Natural Science Foundation of China, U24A20256.

## Ethics Statement

The authors have nothing to report.

## Consent

The authors have nothing to report.

## Conflicts of Interest

The authors declare no conflicts of interest.

## Supporting information


**Data S1:** Detailed datasets and Mendelian randomization (MR) analysis results concerning the causal association between circulating inflammatory cytokines and SOC. This file includes the primary MR results, assessments of heterogeneity and horizontal pleiotropy, MR‐PRESSO analysis, and the specific single nucleotide polymorphisms (SNPs) utilized in the analysis.

**Data S2:** Detailed datasets and Mendelian randomization (MR) analysis results concerning the causal association between metabolites and SOC. This file includes the primary MR results, assessments of heterogeneity and horizontal pleiotropy, MR‐PRESSO analysis, and the specific single nucleotide polymorphisms (SNPs) utilized in the analysis.

**Data S3:** The STROBE‐MR checklist. This document outlines the reporting standards and guidelines followed in this study to ensure transparency and reproducibility.

## Data Availability

The data used in this study are from public available datasets. All data are available through the FinnGen database and NHGRI‐EBI GWAS (https://www.ebi.ac.uk/gwas/).

## References

[1] R. L. Siegel , T. B. Kratzer , A. N. Giaquinto , H. Sung , and A. Jemal , “Cancer Statistics, 2025,” Ca 75, no. 1 (2025): 10–45. 39817679 10.3322/caac.21871 PMC11745215

[2] S. Lheureux , C. Gourley , I. Vergote , and A. M. Oza , “Epithelial Ovarian Cancer,” Lancet (London, England) 393, no. 10177 (2019): 1240–1253. 30910306 10.1016/S0140-6736(18)32552-2

[3] U. A. Matulonis , A. K. Sood , L. Fallowfield , B. E. Howitt , J. Sehouli , and B. Y. Karlan , “Ovarian Cancer,” Nature Reviews. Disease Primers 2 (2016): 16061. 10.1038/nrdp.2016.61 PMC7290868 27558151

[4] K. Tanha , A. Mottaghi , M. Nojomi , et al., “Investigation on Factors Associated With Ovarian Cancer: An Umbrella Review of Systematic Review and Meta‐Analyses,” Journal of Ovarian Research 14 (2021): 153. 34758846 10.1186/s13048-021-00911-z PMC8582179

[5] C. I. Diakos , K. A. Charles , D. C. McMillan , and S. J. Clarke , “Cancer‐Related Inflammation and Treatment Effectiveness,” Lancet. Oncology 15, no. 11 (2014): e493–e503. 25281468 10.1016/S1470-2045(14)70263-3

[6] G. Grant and C. M. Ferrer , “The Role of the Immune Tumor Microenvironment in Shaping Metastatic Dissemination, Dormancy, and Outgrowth,” Trends in Cell Biology (2025). 10.1016/j.tcb.2025.05.006 40628544

[7] G. J. Szebeni , C. Vizler , L. I. Nagy , K. Kitajka , and L. G. Puskas , “Pro‐Tumoral Inflammatory Myeloid Cells as Emerging Therapeutic Targets,” International Journal of Molecular Sciences 17, no. 11 (2016): 1958. 27886105 10.3390/ijms17111958 PMC5133952

[8] G. Hou , Y. Dong , Y. Jiang , et al., “Immune Inflammation and Metabolic Interactions in the Pathogenesis of Diabetic Nephropathy,” Frontiers in Endocrinology 16 (2025): 1602594. 40698245 10.3389/fendo.2025.1602594 PMC12279506

[9] J. Chen , Y. Yang , and W. Kong , “Cross Talk Between Inflammation and Metabolic Disorders,” Mediators of Inflammation 2022 (2022): 9821506. 35462790 10.1155/2022/9821506 PMC9020981

[10] Z. Yun , Z. Guo , X. Li , et al., “Genetically Predicted 486 Blood Metabolites in Relation to Risk of Colorectal Cancer: A Mendelian Randomization Study,” Cancer Medicine 12, no. 12 (2023): 13784–13799. 37132247 10.1002/cam4.6022 PMC10315807

[11] H. Zhong , S. Liu , J. Zhu , T. H. Xu , H. Yu , and L. Wu , “Elucidating the Role of Blood Metabolites on Pancreatic Cancer Risk Using Two‐Sample Mendelian Randomization Analysis,” International Journal of Cancer 154, no. 5 (2024): 852–862. 37860916 10.1002/ijc.34771 PMC10843029

[12] S. Zhang , L. Cao , Z. Li , and D. Qu , “Metabolic Reprogramming Links Chronic Intestinal Inflammation and the Oncogenic Transformation in Colorectal Tumorigenesis,” Cancer Letters 450 (2019): 123–131. 30851417 10.1016/j.canlet.2019.02.045

[13] J. Chen , X. Zhang , R. Cao , et al., “Serum 27‐Nor‐5β‐Cholestane‐3,7,12,24,25 Pentol Glucuronide Discovered by Metabolomics as Potential Diagnostic Biomarker for Epithelium Ovarian Cancer,” Journal of Proteome Research 10, no. 5 (2011): 2625–2632. 21456628 10.1021/pr200173q

[14] O. A. Zeleznik , C. B. Clish , P. Kraft , J. Avila‐Pacheco , A. H. Eliassen , and S. S. Tworoger , “Circulating Lysophosphatidylcholines, Phosphatidylcholines, Ceramides, and Sphingomyelins and Ovarian Cancer Risk: A 23‐Year Prospective Study,” Journal of the National Cancer Institute 112, no. 6 (2020): 628–636. 31593240 10.1093/jnci/djz195 PMC7301073

[15] G. Davey Smith and G. Hemani , “Mendelian Randomization: Genetic Anchors for Causal Inference in Epidemiological Studies,” Human Molecular Genetics 23, no. R1 (2014): R89–R98. 25064373 10.1093/hmg/ddu328 PMC4170722

[16] M. Verduijn , B. Siegerink , K. J. Jager , C. Zoccali , and F. W. Dekker , “Mendelian Randomization: Use of Genetics to Enable Causal Inference in Observational Studies,” Nephrology, Dialysis, Transplantation: Official Publication of the European Dialysis and Transplant Association ‐ European Renal Association 25, no. 5 (2010): 1394–1398. 20190244 10.1093/ndt/gfq098

[17] J. H. Zhao , D. Stacey , N. Eriksson , et al., “Genetics of Circulating Inflammatory Proteins Identifies Drivers of Immune‐Mediated Disease Risk and Therapeutic Targets,” Nature Immunology 24, no. 9 (2023): 1540–1551. 37563310 10.1038/s41590-023-01588-w PMC10457199

[18] Y. Chen , T. Lu , U. Pettersson‐Kymmer , et al., “Genomic Atlas of the Plasma Metabolome Prioritizes Metabolites Implicated in Human Diseases,” Nature Genetics 55, no. 1 (2023): 44–53. 36635386 10.1038/s41588-022-01270-1 PMC7614162

[19] S. S. Savant , S. Sriramkumar , and H. M. O'Hagan , “The Role of Inflammation and Inflammatory Mediators in the Development, Progression, Metastasis, and Chemoresistance of Epithelial Ovarian Cancer,” Cancers 10, no. 8 (2018): 251. 30061485 10.3390/cancers10080251 PMC6116184

[20] Z. Li , N. Huang , W. Zhang , and L. Li , “Biological Function of C‐X‐C Motif Chemokine Ligand 1 Gene (CXCL1) in Ovarian Malignant Tumors[J],” Human & Experimental Toxicology (2023). 10.1177/09603271231203392 37787042

[21] C. Liu , Q. Yin , Z. Wu , et al., “Inflammation and Immune Escape in Ovarian Cancer: Pathways and Therapeutic Opportunities,” Journal of Inflammation Research 18 (2025): 895–909. 39867950 10.2147/JIR.S503479 PMC11762012

[22] K. Li , H. Shi , B. Zhang , et al., “Myeloid‐Derived Suppressor Cells as Immunosuppressive Regulators and Therapeutic Targets in Cancer,” Signal Transduction and Targeted Therapy 6, no. 1 (2021): 362. 34620838 10.1038/s41392-021-00670-9 PMC8497485

[23] B.‐H. Li , M. A. Garstka , and Z.‐F. Li , “Chemokines and Their Receptors Promoting the Recruitment of Myeloid‐Derived Suppressor Cells Into the Tumor,” Molecular Immunology 117 (2020): 201–215. 31835202 10.1016/j.molimm.2019.11.014

[24] M. Yang , G. Zhang , Y. Wang , et al., “Tumour‐Associated Neutrophils Orchestrate Intratumoural IL‐8‐Driven Immune Evasion Through Jagged2 Activation in Ovarian Cancer,” British Journal of Cancer 123, no. 9 (2020): 1404–1416. 32778818 10.1038/s41416-020-1026-0 PMC7591527

[25] M. Nowak , Ł. Janas , M. Soja , et al., “Chemokine Expression in Patients With Ovarian Cancer or Benign Ovarian Tumors,” Archives of Medical Science 18, no. 3 (2021): 682–689. 35591828 10.5114/aoms/110672 PMC9102528

[26] R. Zhang , D. M. Roque , J. Reader , and J. Lin , “Combined Inhibition of IL‐6 and IL‐8 Pathways Suppresses Ovarian Cancer Cell Viability and Migration and Tumor Growth,” International Journal of Oncology 60, no. 5 (2022): 1–9. 35315502 10.3892/ijo.2022.5340 PMC8973967

[27] V. Maggisano , M. D'Amico , S. Aquila , et al., “IL‐20 Subfamily Biological Effects: Mechanistic Insights and Therapeutic Perspectives in Cancer,” International Journal of Molecular Sciences 26, no. 15 (2025): 7320. 40806452 10.3390/ijms26157320 PMC12347730

[28] S. Parveen , M. Fatma , S. S. Mir , S. Dermime , and S. Uddin , “JAK‐STAT Signaling in Autoimmunity and Cancer,” Immuno Targets and Therapy 14 (2025): 523–554. 10.2147/ITT.S485670 PMC12080488 40376194

[29] A. L. Scott , D. E. Jazwinska , D. G. Kulawiec , and I. K. Zervantonakis , “Paracrine Ovarian Cancer Cell‐Derived CSF1 Signaling Regulates Macrophage Migration Dynamics in a 3D Microfluidic Model That Recapitulates In Vivo Infiltration Patterns in Patient‐Derived Xenografts,” Advanced Healthcare Materials 13, no. 28 (2024): e2401719. 38807270 10.1002/adhm.202401719 PMC11560735

[30] S. K. Chambers , “Role of CSF‐1 in Progression of Epithelial Ovarian Cancer,” Future Oncology (London, England) 5, no. 9 (2009): 1429–1440. 19903070 10.2217/fon.09.103 PMC2830097

[31] A. Sehgal , K. M. Irvine , and D. A. Hume , “Functions of Macrophage Colony‐Stimulating Factor (CSF1) in Development, Homeostasis, and Tissue Repair,” Seminars in Immunology 54 (2021): 101509. 34742624 10.1016/j.smim.2021.101509

[32] H. Li and S. Tang , “Colony Stimulating Factor‐1 and Its Receptor in Gastrointestinal Malignant Tumors,” Journal of Cancer 12, no. 23 (2021): 7111–7119. 34729112 10.7150/jca.60379 PMC8558652

[33] O. G. Trifanescu , L. N. Gales , B. C. Tanase , et al., “Prognostic Role of Vascular Endothelial Growth Factor and Correlation With Oxidative Stress Markers in Locally Advanced and Metastatic Ovarian Cancer Patients,” Diagnostics 13, no. 1 (2023): 166. 36611458 10.3390/diagnostics13010166 PMC9818969

[34] X. B. Trinh , W. A. A. Tjalma , P. B. Vermeulen , et al., “The VEGF Pathway and the AKT/mTOR/p70S6K1 Signalling Pathway in Human Epithelial Ovarian Cancer,” British Journal of Cancer 100, no. 6 (2009): 971–978. 19240722 10.1038/sj.bjc.6604921 PMC2661789

[35] C. Lee , M.‐J. Kim , A. Kumar , H.‐W. Lee , Y. Yang , and Y. Kim , “Vascular Endothelial Growth Factor Signaling in Health and Disease: From Molecular Mechanisms to Therapeutic Perspectives,” Signal Transduction and Targeted Therapy 10, no. 1 (2025): 170. 40383803 10.1038/s41392-025-02249-0 PMC12086256

[36] B. L. Krock , N. Skuli , and M. C. Simon , “Hypoxia‐Induced Angiogenesis: Good and Evil,” Genes & Cancer 2, no. 12 (2011): 1117–1133. 22866203 10.1177/1947601911423654 PMC3411127

[37] P. Carmeliet and R. K. Jain , “Molecular Mechanisms and Clinical Applications of Angiogenesis,” Nature 473, no. 7347 (2011): 298–307. 21593862 10.1038/nature10144 PMC4049445

[38] W. A. Spannuth , A. M. Nick , N. B. Jennings , et al., “Functional Significance of VEGFR‐2 on Ovarian Cancer Cells,” International Journal of Cancer. Journal International du Cancer 124, no. 5 (2009): 1045–1053. 19058181 10.1002/ijc.24028 PMC2668132

[39] I. Sher , S. A. Adham , J. Petrik , and B. L. Coomber , “Autocrine VEGF‐A/KDR Loop Protects Epithelial Ovarian Carcinoma Cells From Anoikis,” International Journal of Cancer 124, no. 3 (2009): 553–561. 19004006 10.1002/ijc.23963

[40] B.‐Q. Guo and W.‐Q. Lu , “The Prognostic Significance of High/Positive Expression of Tissue VEGF in Ovarian Cancer,” Oncotarget 9, no. 55 (2018): 30552–30560. 30093968 10.18632/oncotarget.25702 PMC6078137

[41] Q. Cai , Z. Zhao , C. Antalis , et al., “Elevated and Secreted Phospholipase A2 Activities as New Potential Therapeutic Targets in Human Epithelial Ovarian Cancer,” FASEB Journal 26, no. 8 (2012): 3306–3320. 22767227 10.1096/fj.12-207597 PMC3405265

[42] S. G. Tewari , R. P. Swift , J. Reifman , S. T. Prigge , and A. Wallqvist , “Metabolic Alterations in the Erythrocyte During Blood‐Stage Development of the Malaria Parasite,” Malaria Journal 19 (2020): 94. 32103749 10.1186/s12936-020-03174-z PMC7045481

[43] X. Fang , S. Yu , R. C. Bast , et al., “Mechanisms for Lysophosphatidic Acid‐Induced Cytokine Production in Ovarian Cancer Cells,” Journal of Biological Chemistry 279, no. 10 (2004): 9653–9661. 14670967 10.1074/jbc.M306662200

[44] X. Wang , Y.‐F. Li , G. Nanayakkara , et al., “Lysophospholipid Receptors, as Novel Conditional Danger Receptors and Homeostatic Receptors Modulate Inflammation ‐ Novel Paradigm and Therapeutic Potential,” Journal of Cardiovascular Translational Research 9, no. 4 (2016): 343–359. 27230673 10.1007/s12265-016-9700-6 PMC4992420

[45] N. Shen , S. Lu , Z. Kong , et al., “The Causal Role Between Circulating Immune Cells and Diabetic Nephropathy: A Bidirectional Mendelian Randomization With Mediating Insights,” Diabetology & Metabolic Syndrome 16 (2024): 164. 39014501 10.1186/s13098-024-01386-w PMC11253417

