# Supplementary material for: Causal Interplay Between Inflammatory Cytokines and Lipid Metabolites in Serous Ovarian Carcinoma: Insights From a Genetic Association Study
Source: J Clin Lab Anal. 2026 May 8;40(12):e70250. doi: 10.1002/jcla.70250 (PMC13327467; doi:10.1002/jcla.70250)
Supplement: Supplementary file 3 — Data S3: The STROBE‐MR checklist. This document outlines the reporting standards and guidelines followed in this study to ensure transparency and reproducibility. [file JCLA-40-e70250-s003.docx]

**STROBE-MR checklist of recommended items to address in reports of Mendelian randomization studies**^1^ ^2^

| **Item No.** | **Section** | **Checklist item** | **Page No.** | **Relevant text from manuscript** |
| --- | --- | --- | --- | --- |
| 1 | **TITLE and ABSTRACT** | Indicate Mendelian randomization (MR) as the study’s design in the title and/or the abstract if that is a main purpose of the study | 1-2 | Blood metabolites mediate the association between circulating inflammatory proteins and serous ovarian cancer: a two-step, two-sample Mendelian randomization study |
|  | **INTRODUCTION** |  |  |  |
| 2 | **Background** | Explain the scientific background and rationale for the reported study. What is the exposure? Is a potential causal relationship between exposure and outcome plausible? Justify why MR is a helpful method to address the study question | 3-4 | Ovarian cancer remains a leading cause of tumor-related mortality among women worldwide. Among its histological subtypes, serous ovarian carcinoma (SOC)—particularly high-grade serous ovarian carcinoma (HGSOC)…FinnGen and the NHGRI-EBI GWAS catalog, the feasibility and reproducibility of these strategies are now greatly enhanced. |
| 3 | **Objectives** | State specific objectives clearly, including pre-specified causal hypotheses (if any). State that MR is a method that, under specific assumptions, intends to estimate causal effects | 4 | Building on these considerations, the present study was designed to test two hypotheses…while laying the foundation for subsequent functional validation and translational research. |
|  | **METHODS** |  |  |  |
| 4 | **Study design and data sources** | Present key elements of the study design early in the article. Consider including a table listing sources of data for all phases of the study. For each data source contributing to the analysis, describe the following: | 4 | In this study, we employed a two-step MR approach to investigate the potential causal associations between inflammatory factors and SOC…and subsequently quantified the mediating effects of metabolites on the inflammatory factor–SOC pathway (Figure 1). |
|  | a) | Setting: Describe the study design and the underlying population, if possible. Describe the setting, locations, and relevant dates, including periods of recruitment, exposure, follow-up, and data collection, when available. | 4 | Genome-wide association study (GWAS) summary data for ovarian cancer were obtained from the FinnGen consortium …were downloaded from the NHGRI-EBI GWAS Catalog (https://www.ebi.ac.uk/gwas/). |
|  | b) | Participants: Give the eligibility criteria, and the sources and methods of selection of participants. Report the sample size, and whether any power or sample size calculations were carried out prior to the main analysis | 4 |  |
|  | c) | Describe measurement, quality control and selection of genetic variants | 5 | Single nucleotide polymorphisms (SNPs) significantly associated with the exposures of interest were selected as instrumental variables (IVs)…regarded as weak instruments and excluded from subsequent analyses. |
|  | d) | For each exposure, outcome, and other relevant variables, describe methods of assessment and diagnostic criteria for diseases | 10 | The data used in this study are from public available datasets. All data are available through the FinnGen database and NHGRI-EBI GWAS (https://www.ebi.ac.uk/gwas/). |
|  | e) | Provide details of ethics committee approval and participant informed consent, if relevant | 4 | All data used in this study were obtained from publicly available datasets; therefore, no additional ethical approval or informed consent was required. |
| 5 | **Assumptions** | Explicitly state the three core IV assumptions for the main analysis (relevance, independence and exclusion restriction) as well assumptions for any additional or sensitivity analysis | 4 | The primary analytic strategy of this study was two-sample MR. MR analysis relies on three core assumptions to ensure unbiased causal inference: …no additional ethical approval or informed consent was required. |
| 6 | **Statistical methods: main analysis** | Describe statistical methods and statistics used | 5 | Statistical analysis |
|  | a) | Describe how quantitative variables were handled in the analyses (i.e., scale, units, model) | 5 |  |
|  | b) | Describe how genetic variants were handled in the analyses and, if applicable, how their weights were selected | 5 | Results were reported as odds ratios (ORs) with corresponding 95% confidence intervals (CIs), and a P-value < 0.05 was considered statistically significant. |
|  | c) | Describe the MR estimator (e.g. two-stage least squares, Wald ratio) and related statistics. Detail the included covariates and, in case of two-sample MR, whether the same covariate set was used for adjustment in the two samples | 6 | MR analyses were performed using five methods: inverse-variance weighted (IVW), MR-Egger, weighted median, simple mode, and weighted mode. Among these, IVW was considered the primary analytic approach |
|  | d) | Explain how missing data were addressed | none |  |
|  | e) | If applicable, indicate how multiple testing was addressed | none |  |
| 7 | **Assessment of assumptions** | Describe any methods or prior knowledge used to assess the assumptions or justify their validity | 4 | The primary analytic strategy of this study was two-sample MR. MR analysis relies on three core assumptions to ensure unbiased causal inference: …no additional ethical approval or informed consent was required. |
| 8 | **Sensitivity analyses and additional analyses** | Describe any sensitivity analyses or additional analyses performed (e.g. comparison of effect estimates from different approaches, independent replication, bias analytic techniques, validation of instruments, simulations) | 5 | To further ensure the robustness of causal inferences in the two-sample MR framework… If the overall confidence interval showed no substantial changes after removing individual SNPs, the result was considered robust. |
| 9 | **Software and pre-registration** |  |  |  |
|  | a) | Name statistical software and package(s), including version and settings used | 7 | All statistical analyses were performed using R software (version 4.2.1), with the TwoSampleMR package applied for MR analyses. |
|  | b) | State whether the study protocol and details were pre-registered (as well as when and where) | none |  |
|  | **RESULTS** |  |  |  |
| 10 | **Descriptive data** |  |  |  |
|  | a) | Report the numbers of individuals at each stage of included studies and reasons for exclusion. Consider use of a flow diagram | 6 | Through MR analysis, we identified five inflammatory factors and 68 blood metabolites with potential causal associations with SOC. Mediation MR analysis further revealed that 1-palmitoyl-GPG (16:0) mediated the effect of IL-8 on SOC (Supplementary Materials 1–2). |
|  | b) | Report summary statistics for phenotypic exposure(s), outcome(s), and other relevant variables (e.g. means, SDs, proportions) | 6 | Effect of Inflammatory Factors on SOC  Effect of Blood Metabolites on SOC  Effect of Inflammatory Factors on Blood Metabolites  Mediation effect of 1-Palmitoyl-GPG (16:0) in the causal association between the IL8 and SOC |
|  | c) | If the data sources include meta-analyses of previous studies, provide the assessments of heterogeneity across these studies | none |  |
|  | d) | For two-sample MR:  i.  Provide justification of the similarity of the genetic variant-exposure associations between the exposure and outcome samples  ii.  Provide information on the number of individuals who overlap between the exposure and outcome studies | 6 | (Supplementary Materials 1–2). |
| 11 | **Main results** |  |  |  |
|  | a) | Report the associations between genetic variant and exposure, and between genetic variant and outcome, preferably on an interpretable scale | 6 | (Supplementary Materials 1–2). |
|  | b) | Report MR estimates of the relationship between exposure and outcome, and the measures of uncertainty from the MR analysis, on an interpretable scale, such as odds ratio or relative risk per SD difference | 6 | (Supplementary Materials 1–2). |
|  | c) | If relevant, consider translating estimates of relative risk into absolute risk for a meaningful time period | 6 | IVW analyses identified five inflammatory factors with significant causal associations with SOC… VEGF-A (OR = 1.239, 95% CI: 1.000–1.535) (Figures 2 and 3). |
|  | d) | Consider plots to visualize results (e.g. forest plot, scatterplot of associations between genetic variants and outcome versus between genetic variants and exposure) | 14-15 | Figure 2-4 |
| 12 | **Assessment of assumptions** |  |  | Cochran’s Q test indicated no significant heterogeneity among the selected SNPs. Neither the MR-Egger regression nor the MR-PRESSO global test detected evidence of horizontal pleiotropy. Furthermore, leave-one-out analysis demonstrated that the observed causal relationships were not driven by any single SNP. |
|  | a) | Report the assessment of the validity of the assumptions | 6 |  |
|  | b) | Report any additional statistics (e.g., assessments of heterogeneity across genetic variants, such as *I^2^*, Q statistic or E-value) | 6 |  |
| 13 | **Sensitivity analyses and additional analyses** |  |  | Cochran’s Q test indicated no significant heterogeneity among the selected SNPs. Neither the MR-Egger regression nor the MR-PRESSO global test detected evidence of horizontal pleiotropy. Furthermore, leave-one-out analysis demonstrated that the observed causal relationships were not driven by any single SNP. |
|  | a) | Report any sensitivity analyses to assess the robustness of the main results to violations of the assumptions | 6 |  |
|  | b) | Report results from other sensitivity analyses or additional analyses | 6 |  |
|  | c) | Report any assessment of direction of causal relationship (e.g., bidirectional MR) | 6 |  |
|  | d) | When relevant, report and compare with estimates from non-MR analyses | none |  |
|  | e) | Consider additional plots to visualize results (e.g., leave-one-out analyses) | 9 | Supplementary Material 1. Circulating inflammatory factors to SOC  Supplementary Material 2. Metabolites to SOC |
|  | **DISCUSSION** |  |  |  |
| 14 | **Key results** | Summarize key results with reference to study objectives | 7 | In this study, we applied bidirectional MR to screen 91 inflammatory factors and identified five with potential causal associations with SOC. Furthermore, we employed two-step mediation MR to examine whether blood metabolites mediated the effects of inflammatory factors on SOC. |
| 15 | **Limitations** | Discuss limitations of the study, taking into account the validity of the IV assumptions, other sources of potential bias, and imprecision. Discuss both direction and magnitude of any potential bias and any efforts to address them | 8 | Nevertheless, several limitations should be acknowledged. First, the datasets employed in this study were primarily derived from European populations; |
| 16 | **Interpretation** |  |  |  |
|  | a) | Meaning: Give a cautious overall interpretation of results in the context of their limitations and in comparison with other studies | 8 | Collectively, our findings provide evidence that inflammatory factors may exert causal roles in the initiation and progression of SOC, with blood metabolites mediating part of these effects. This discovery offers novel molecular insights into the etiology of SOC, supporting the identification and development of more targeted biomarkers, and laying the foundation for new therapeutic strategies directed at inflammation, immunity, metabolism, and angiogenesis, thereby advancing personalized and precision medicine for SOC. |
|  | b) | Mechanism: Discuss underlying biological mechanisms that could drive a potential causal relationship between the investigated exposure and the outcome, and whether the gene-environment equivalence assumption is reasonable. Use causal language carefully, clarifying that IV estimates may provide causal effects only under certain assumptions | 9- | Chronic inflammation and immune imbalance constitute a central background for the initiation and progression of SOC[19]……. |
|  | c) | Clinical relevance: Discuss whether the results have clinical or public policy relevance, and to what extent they inform effect sizes of possible interventions | 9- | Chronic inflammation and immune imbalance constitute a central background for the initiation and progression of SOC[19]……. |
| 17 | **Generalizability** | Discuss the generalizability of the study results (a) to other populations, (b) across other exposure periods/timings, and (c) across other levels of exposure | 9 | First, the datasets employed in this study were primarily derived from European populations; thus, the generalizability of our results to other ancestries requires further validation. |
|  | **OTHER INFORMATION** |  |  |  |
| 18 | **Funding** | Describe sources of funding and the role of funders in the present study and, if applicable, sources of funding for the databases and original study or studies on which the present study is based | 9 | Not applicable. |
| 19 | **Data and data sharing** | Provide the data used to perform all analyses or report where and how the data can be accessed, and reference these sources in the article. Provide the statistical code needed to reproduce the results in the article, or report whether the code is publicly accessible and if so, where | 10 | The data used in this study are from public available datasets. All data are available through the FinnGen database and NHGRI-EBI GWAS (https://www.ebi.ac.uk/gwas/). |
| 20 | **Conflicts of Interest** | All authors should declare all potential conflicts of interest | 10 | The authors declare no conflict of interest. |

This checklist is copyrighted by the Equator Network under the Creative Commons Attribution 3.0 Unported (CC BY 3.0) license.

1. Skrivankova VW, Richmond RC, Woolf BAR, Yarmolinsky J, Davies NM, Swanson SA, et al. Strengthening the Reporting of Observational Studies in Epidemiology using Mendelian Randomization (STROBE-MR) Statement. JAMA. 2021;under review.

2. Skrivankova VW, Richmond RC, Woolf BAR, Davies NM, Swanson SA, VanderWeele TJ, et al. Strengthening the Reporting of Observational Studies in Epidemiology using Mendelian Randomisation (STROBE-MR): Explanation and Elaboration. BMJ. 2021;375:n2233.
